# ‘I Am a Person Before My Label’: Exploring People's Perceptions of Different Discourses Associated With Dyslexia

**DOI:** 10.1002/dys.70052

**Published:** 2026-10-25

**Authors:** Stephen J. Macdonald, Amy Pearson, Monique Botha, Jennifer Leigh, Kathryn Williams, Gemma Williams, Elliott Spaeth, Kieran Rose, Ann Memmott, Aimee Grant

**Affiliations:** ^1^ Department of Sociology Durham University Durham UK; ^2^ Department of Psychology Durham University Durham UK; ^3^ School of Social Sciences Kent University Kent UK; ^4^ School of Health and Social Care Swansea University Swansea UK; ^5^ School of Psychology, Sport and Health Sciences University of Portsmouth Portsmouth UK; ^6^ The Autistic Advocate Essex UK; ^7^ AM Consulting Berkshire UK; ^8^ The School of Health and Social Care Swansea University Swansea UK

## Abstract

Language plays a central role in shaping how dyslexia is understood and negotiated across education, employment and everyday life. While debates have historically focused on diagnosis, less attention has been given to how adults who identify as dyslexic interpret and evaluate the labels applied to them. This article examines dyslexic adults' perceptions of dyslexia‐related and neurodiversity movement terminology, drawing on a UK mixed‐methods survey of 166 participants who identified as dyslexic, with or without co‐occurring neurodivergences. Quantitative findings reveal a clear hierarchy of label preference. Participants strongly favoured dyslexia‐specific, identity‐based terms such as ‘dyslexic’, while rejecting broader, deficit‐centred labels including ‘learning difficulty’, ‘specific learning disability’ and ‘specific learning disorder’. Qualitative analysis shows these preferences are shaped by experiences of stigma and misrecognition. The findings show that adoption of a neurodivergent label varies depending on whether participants have single or multiple neurodivergent identities. Those with dyslexia alone expressed greater ambivalence towards the label of neurodiversity. In contrast, dyslexic participants alongside autism or ADHD were more likely to adopt the label. The study highlights the importance of listening to dyslexic voices in debates about terminology. It cautions against deficit‐based and overly generalised language which risks reproducing stigma rather than challenging oppression.

## Points of Interest

1. Dyslexic adults strongly prefer dyslexia‐specific labels, particularly the term dyslexic.
2. Deficit‐based terms such as learning difficulty, disability, and disorder were widely rejected
3. Language shapes identity and recognition in the lives of dyslexic adults
4. Neurodivergent identity was unevenly adopted, especially among dyslexic participants.
5. Debates about terminology should centre on the voices of dyslexic people.

## 1 Introduction

Dyslexia has long occupied a contested position within education, psychology and disability politics (Campbell 2013). It has been framed as a neurological defect, specific learning difficulty, cognitive difference and, more recently, as part of neurodiversity discourse (Odegard and Dye 2024). These shifting conceptualisations have generated debate over definition, diagnosis and legitimacy, often prioritising scientific classification over lived experience (Campbell 2013; Macdonald 2013). Yet language does not simply describe dyslexia; it shapes how dyslexic people are understood, valued and treated within institutions and everyday interactions. Language structures access to support, signals legitimacy or deficiency and mediates identity formation across the life course. Within disability studies, language is recognised as a key site of political struggle (Oliver 2009; Barnes 2019). Labels can function as instruments of oppression or as tools for mobilisation. At the same time, critical debates caution against homogenising identities or reproducing deficit‐based framings through diagnostic categories (Campbell 2013; Bone 2017). These tensions are particularly pronounced in relation to dyslexia, where stigma often predates diagnosis and is tied to assumptions about intelligence and competence (Macdonald 2010, 2013; Kirby 2020). For many dyslexic people, labels are practical resources. They are used to explain needs, resist misrecognition and negotiate inclusion within disabling environments (Macdonald 2013).

The neurodiversity movement has emerged as a counter‐discourse to medicalised understandings of cognitive difference (Chapman and Carel 2022). While challenging deficit framings, it raises questions about identity, specificity and inclusion (Ne'eman and Pellicano 2022). Disability studies warn that umbrella terms risk obscuring inequalities or privileging certain forms of difference (Runswick‐Cole 2014). Empirical research exploring how dyslexic adults evaluate such labels remains limited, with most scholarship focusing on professional debates concerning labelling (Snowling et al. 2020). This article addresses that gap by examining dyslexic adults' preferences for dyslexia‐related and neurodiversity‐related terms. Drawing on a UK mixed‐methods survey, it explores how labels are experienced as empowering, stigmatising, inaccurate or strategically useful and how these evaluations vary by diagnostic profile.

## 2 Shifting Discourses: From Congenital Word‐Blindness to Developmental Dyslexia

The history of dyslexia is marked by shifting labels and conceptual frameworks that reflect broader changes in medical, psychological, educational and political understandings of reading and writing difficulties (Campbell 2013; Kirby 2020). Early accounts located reading problems within neurology, using concepts such as ‘aphasia’ and ‘alexia’ to position them within broader language disorders (Campbell 2013). The term ‘word‐blindness’ emerged to describe individuals who struggled to read despite otherwise typical intelligence and development, distinguishing reading difficulty from intellectual impairment. Initially understood as acquired, reading difficulties were subsequently recognised in developmental forms (Campbell 2013; Kirby 2020). Over the twentieth century, explanations increasingly shifted from visual towards language‐based accounts, and ‘developmental dyslexia’ became an established term (Campbell 2013; Kirby 2020). Growing political recognition was reflected in the establishment of the British Dyslexia Association, although disputes persisted over whether the dyslexia label obscured social and educational factors contributing to reading difficulties (Kirby 2019, 2020).

By the late twentieth century, psycholinguistic models had become influential, framing dyslexia as a language‐based ‘specific learning difficulty’ centred on phonological processing (Campbell 2013; Kirby 2020). Contemporary classifications include ‘specific learning difficulty’ and ‘specific learning disorder’, reflecting the continuing influence of cognitive and deficit‐based explanations within education and psychology. Debates over the legitimacy and value of dyslexia as a category remain prominent (Elliott and Grigorenko 2024), shaping struggles for recognition and inclusion (Macdonald 2010, 2013). Political advocacy has also secured recognition within equality and disability legislation, enabling access to support (Kirby 2020). More recently, neurodiversity perspectives have positioned dyslexia as a form of neurological variation (Macdonald 2010, 2019). These shifts demonstrate how terminology evolves alongside scientific, educational and political change, shaping recognition, support and social meaning. Historically, terminology has largely been produced within medical and educational institutions, with limited input from the dyslexic community. The history of dyslexia is therefore not simply a history of scientific progress, but a record of ongoing struggles over meaning, labelling, legitimacy and inclusion.

## 3 The Disability Movement and Identity Politics

The history of the UK disability movement is central to debates about identity, political recognition and the role of labelling. From its emergence in the 1970s, the movement developed in opposition to medicalised understandings of disability that framed impairment as individual tragedy or deficit (Oliver 2009). Activists instead redefined disability as a socially produced form of oppression rooted in environmental, institutional and attitudinal barriers. This shift laid the groundwork for a collective disability identity that was explicitly political, with labelling functioning not merely as description but as a mechanism for mobilisation and resistance (Barnes 2019). Central to this process was the development of the social model of disability, which distinguished between impairment and disability. Impairment referred to bodily or cognitive difference, while disability was defined as the exclusionary nature of social structures that include or exclude disabled people from participation across the life course (Oliver 2009; Macdonald 2019). This reframing enabled disabled people to challenge dominant medical labels and assert a shared political identity grounded in opposition to structural oppression (Barnes 2019). Labelling became strategically important in facilitating collective action, access to rights‐based frameworks and campaigns for legislative and social change. It was from this movement that identity‐first terminology emerged, with disabled activists embracing the term ‘disabled people’ to describe people disabled by society and rejecting person‐first language associated with medical discourse (Oliver 2009).

However, the emergence of disability identity politics has attracted critique within disability studies. Shakespeare (2013) highlights the risks of assuming a disabled identity based solely on social exclusion, arguing that the social model neglects the impact of impairment (Shakespeare and Watson 2023). From this perspective, impairment, understood as difference, interacts with disability at the structural level and must be incorporated into people's experiences (Shakespeare 2013; Shakespeare and Watson 2023). While identity politics were crucial in challenging exclusion, basing identity entirely on disability overlooks the complexity of how identities are formed through both social oppression and lived experiences of impairment (Shakespeare 2013). These tensions are particularly evident in debates concerning neurodiversity and dyslexia. Riddick (2000) and Macdonald (2010) challenge ‘anti‐labelling’ approaches that reject diagnostic categories as inherently stigmatising, arguing that medicalisation has been oversimplified. Being medically labelled can increase access to resources and inclusive practices, while stigma often precedes diagnosis and is rooted in assumptions of low intelligence rather than the dyslexia label itself (Riddick 2000; Macdonald 2010, 2013, 2019; Odegard and Dye 2024). Research applying the social model to dyslexia suggests that impairment‐specific labels, like dyslexia, can be necessary to recognise and confront experiences of exclusion and discrimination (Riddick 2000; Odegard and Dye 2024). Identity politics are therefore about control over meaning, resources, and representations, as well as capturing lived experience. Labels have been central in forging collective identities and securing legal recognition (Barnes 2019), yet they remain provisional and contested, shaped by power relations produced through medical research and academia. The challenge is to retain the political importance of identity without over simplifying the lived experience of being disabled (Shakespeare 2013).

## 4 The Politics of the Neurodiversity Movement

Emerging from the disability rights movement and autism activism of the 1990s, the neurodiversity movement has shaped contemporary debates around neurodivergent identities such as autism, ADHD and, to a lesser extent, dyslexia (Macdonald 2019; Chapman and Carel 2022). The politics of neurodiversity emerged from activist challenges to medicalised and deficit‐based understandings of cognitive difference (Chapman and Carel 2022) and have developed into a contested field of political struggle. Neurodiversity is not simply a descriptive term defining neurological variation; it is a political concept that raises questions about identity, diagnosis, disability, power and social justice (Chapman and Carel 2022; Ne'eman and Pellicano 2022). Debates over its meaning, scope and ownership reveal tensions between activism, academia, policy and neurodivergent communities. Botha et al. (2024) demonstrate that the neurodiversity movement did not emerge from a single theorist but developed from multiple sources across different contexts, including online spaces in the mid‐1990s prior to its academic formulation by Judy Singer. This collective origin highlights how the neurodiversity movement emerged through activist struggle, mutual recognition, and resistance to pathologisation (Chapman and Carel 2022; Botha et al. 2024).

Ne'eman and Pellicano (2022) emphasise that neurodiversity should be understood as a social justice movement rather than a diagnostic category. From this perspective, the neurodiversity movement is concerned with power relations, human rights and the redistribution of authority over professional knowledge. Central to this politics is a critique of professional dominance within medicine, psychology, and education, which historically defined neurodivergences through deficit discourses. The neurodiversity movement challenges these practices by asserting the importance of lived experience and reframing difference as a natural aspect of human variation. Chapman and Carel (2022) argue that neurodivergent people are systematically marginalised as knowers, with their testimonies discounted through medical authority. Neurodiversity politics therefore seeks to value neurodivergent perspectives and address whose knowledge counts. However, the political coherence of the neurodiversity movement has been controversial. Jones and Orchard (2024) warn that rejecting disability within the neurodiversity movement risks obscuring structural disadvantage and weakening access to legal protections and collective political struggle. Runswick‐Cole (2014) highlights how neurodiversity operates within neoliberal contexts that celebrate difference while intensifying inequality, producing an ‘us/them’ binary between valued and less valued neurodivergent subjects. In this context, difference may be recognised only where it aligns with productivity, employability and individual resilience. This creates tension between neurodiversity as a collective politics and its uptake as an individualised identity.

Beck (2023) further argues that when neurodiversity is reduced to personal branding or self‐expression, its political potential is undermined. Instead, neurodiversity should be understood through collective practices, mutual care and alternative ways of knowing. Walters (2025) similarly shows how neurodiversity has become entangled with policy, education and welfare regimes, often reinforcing existing power structures. While it enables new forms of recognition, it has also been incorporated into managerial frameworks that prioritise inclusion without producing structural change (Beck 2023; Walters 2025). Critiques of the neurodiversity movement have also emerged from dyslexia studies. Although the neurodiversity movement has shaped research on autism and ADHD, dyslexia is often overlooked. The concept began to emerge within dyslexia studies in the mid‐2000s, largely through applications of the social model (Pollak 2009; Macdonald 2019). While the neurodiversity movement offers a counter‐narrative to deficit models, particularly where experiences are shaped by stigma (Adam‐Bagley 2022; Odegard and Dye 2024), rejecting the discourse of disability may have unintended consequences, including exclusion from support (Jones and Orchard 2024). This is evident in the education system, which has moved away from dyslexia diagnosis towards classrooms that claim to include all neurodivergent children but may include none. From this perspective, accepting or rejecting labels is a political act (Macdonald 2013), shaping meaning, inequality and representation. Without the consent of neurodivergent communities, such shifts in discourses risk undermining the aims of the neurodiversity movement to represent lived experience.

## 5 Methodology

### 5.1 Study Design

This study adopted a cross‐sectional mixed‐methods design, combining quantitative analysis with qualitative reflexive thematic analysis. The article is reported in line with the Consensus‐Based Checklist for Reporting of Survey Studies (CROSS) (Sharma et al. 2021). The study aimed to explore the views and experiences of neurodiversity‐related terminology among adults in the UK who identify as dyslexic. While the wider survey (see Grant et al. 2025; Pearson et al. 2026) included participants with a range of neurodevelopmental neurodivergences, the present analysis focuses on the subsample of dyslexic participants (*n* = 166), with or without co‐occurring neurodivergences.

### 5.2 Researcher Positionality

The research team comprises neurodivergent researchers based in the UK with expertise in neurodivergence and disability studies. Collectively, the authors represent a range of neurodivergent identities, including dyslexia, alongside varied professional backgrounds and lived experiences. These include physical and sensory impairments, semi‐speaking identities, different genders and sexualities and ethnic minority backgrounds. The authors' approach was informed by disability studies and the neurodiversity movement, particularly their emphasis on the value of lived experience and their questioning of professional authority in defining disability and neurodivergence. This positioning informed a critical engagement with terminology, identity and power, while remaining attentive to participants' differing relationships with disability and neurodivergent identities. These positionalities informed the analytical process and interpretation of findings, particularly in relation to identity, stigma and language.

### 5.3 Participants and Recruitment

Participants were eligible if they were aged 18 years or over, lived in the UK and identified as having at least one form of neurodivergence. Both formally diagnosed individuals and those who self‐identified were eligible to participate. Neurodivergent identities included dyslexia, ADHD, autism, dyspraxia, dyscalculia, dysgraphia, developmental language conditions, sensory and processing differences, Tourette syndrome or tics and related neurodivergences. The present article focuses on the 166 participants who identified as dyslexic, enabling exploration of dyslexia‐specific experiences of terminology within this broader neurodivergent context. Participants were recruited using convenience and snowball sampling. The survey was disseminated through neurodivergence‐related organisations, including third‐sector support groups, as well as researchers' networks and social media platforms, primarily Twitter and Facebook. Data collection took place between May and September 2022.

### 5.4 Participant Characteristics

The analytical sample comprised 166 participants (see Table 1). Most participants identified as female (75.3%, *n* = 122), with smaller groups identifying as male (14.2%, *n* = 23) or non‐binary (10.5%, *n* = 17). The age profile was concentrated in early to mid‐adulthood, with the largest proportions aged 25–34 (31.7%, *n* = 52) and 35–44 (32.3%, *n* = 53). Fewer participants were aged 18–24 (11.6%, *n* = 19) or over 55 (7.3% combined, *n* = 12). Employment status varied. A majority were in paid employment, either full‐time (38.4%) or part‐time (18.3%). Other participants reported being unable to work (12.8%, *n* = 21), students (11.0%, *n* = 18), unemployed (6.1%, *n* = 10), self‐employed (6.7%), retired (3.0%) or carers (3.7%). Nearly three‐quarters held an undergraduate degree or higher: 39.8% (*n* = 66) reported an undergraduate degree, 25.3% (*n* = 42) a taught postgraduate qualification, and 7.2% (*n* = 12) a doctorate. Smaller proportions reported no qualifications (1.8%) or GCSEs only (5.4%). The sample was predominantly White (95.2%, *n* = 158), with small numbers identifying as Mixed or from other ethnic groups. Most participants resided in England (81.3%), followed by Scotland (12.0%), Wales (6.0%) and Northern Ireland (0.6%).

**TABLE 1:** Social demographics.

Characteristics	Percent	*n*
Gender	100%	162
Male	14.2%	23
Female	75.3%	122
Non‐binary	10.5%	17
Age	100%	164
18–24	11.6	19
25–34	31.7	52
35–44	32.3	53
45–54	17.1	28
55–64	6.7	11
65+	0.6	1
Employment	100%	164
Employed full time	38.4	63
Employed part time	18.3	30
Self employed	6.7	11
Unemployed	6.1	10
Retired	3.0	5
Student	11.0	18
Unable to work	12.8	21
Carer	3.7	6
Ethnic groups	100%	166
White	95.2	158
Mixed or ethnic groups	3.6	6
Asian or Asian British	0.6	1
Other	0.6	1
Current residence	100%	
England	81.3	166
Scotland	12.0	135
Wales	6.0	20
Northern Ireland	0.6	10
Educational achievements	100%	166
None	1.8	3
GCSEs or equivalent	5.4	9
A levels or equivalent	7.8	13
NVQ or equivalent	6.6	11
Undergraduate degree	39.8	66
Taught postgraduate degree	25.3	42
Doctorate (PhD)	7.2	12
Other	6.0	10

*Note:* *n*, participant numbers.

### 5.5 Data Collection

Data were collected via an online survey hosted on Qualtrics. Participation was voluntary, and no questions were mandatory. First, demographic information was collected. Second, participants were asked closed and open‐ended questions relating to each developmental neurodivergence they reported identifying with (see authors, 2026). Third, the survey included two closed questions and three open‐ended questions focused specifically on neurodiversity‐related terminology (see authors, 2025). Participants were then invited to respond in free‐text format to four further open‐ended questions, including reflections on the language used in the survey and its impact on their life course.

### 5.6 Ethical Considerations

Ethical approval was granted by the University of Sunderland Research Ethics Committee approved the study (reference: 010358). Participants accessed an online information sheet and provided informed consent electronically before participation. Confidentiality and anonymity were assured, and identifying details were removed from all outputs. Participants could skip any question or withdraw at any point.

### 5.7 Data Analysis

Data relating to participants who identified as dyslexic (*n* = 166) were extracted from the wider dataset and analysed separately for this paper.

### 5.8 Quantitative Analysis

Quantitative data were analysed using SPSS (versions 27 and 29), following established approaches to survey and categorical data (De Vaus 2014). Given the predominance of nominal and ordinal variables, analysis focused on descriptive statistics and bivariate associations rather than predictive modelling. Univariate analysis examined frequency distributions, followed by cross‐tabulations to explore relationships between variables. Statistical significance was assessed using chi‐square tests or Fisher's exact test where required, with *p* ≤ 0.05. Only significant data were included in this paper.

### 5.9 Qualitative Analysis

Responses to the open‐ended questions were analysed using reflexive thematic analysis (Braun and Clarke 2021), supported by NVivo. The first author conducted the analysis following the phases outlined by Braun and Clarke (2021), including data familiarisation, systematic coding and the development and refinement of themes. Coding was conducted inductively, with themes iteratively developed through close engagement with the data. The resulting analysis was written up and shared with co‐authors for feedback and critical reflection.

## 6 Findings: Dyslexia and Neurodiversity

Within the literature, debates concerning labelling are often linked to diagnosis. From a disability studies perspective, identity emerges through medicalisation (Barnes 2019). However, from a neurodiversity perspective, neurodivergent identity can emerge through self‐identification (Chapman and Carel 2022). This was explored in the data analysis, specifically whether most participants were formally diagnosed with dyslexia or whether they self‐identified (see Table 2). Within the data analysis, the majority of dyslexic participants reported having a clinical diagnosis (84.9%, *n* = 141), while a smaller proportion self‐identified (10.8%, *n* = 18) or were undergoing assessment at the time of the study (3.0%, *n* = 5). This suggests that the majority of participants reported being diagnosed with dyslexia rather than self‐identification. Participants were also asked about the impact of neurodiversity, disability or mental health on their daily activities (see Table 2). Over four‐fifths reported some level of impact, with just over half indicating that it affected them ‘a little’ (50.6%, *n* = 84) and a further third reporting that it affected them ‘a lot’ (33.1%, *n* = 55). Only 16.3% (*n* = 27) reported no impact at all. These findings indicate that, for most participants, dyslexia and related neurodivergences were experienced as having ongoing consequences on their daily experiences, which is highly relevant when considering why particular labels may be preferred or resisted.

**TABLE 2:** Dyslexic participants' diagnostic status and reported impact of neurodiversity, disability or mental health on daily activities.

Neurodiversity	Percent	*n*
Dyslexia diagnosis/assessment	100%	166
Clinically diagnosed	84.9	141
Self‐diagnosed	10.8	18
Undergoing assessment	3.0	5
Prefer not to say	1.2	2
Impact of neurodiversity/disability/mental health on daily activities	100%	166
Yes, a little	50.6	84
Yes, a lot	33.1	55
Not at all	16.3	27

*Note:* *n*, participant numbers.

Within the neurodiversity literature, alongside current educational research, some scholars argue that dyslexia is extremely difficult to isolate as a distinct neurodivergence (Brimo et al. 2021). For most people, there is a degree of co‐occurrence with other neurodivergent identities (Brimo et al. 2021). To explore this, we asked whether participants identified with other neurodivergent identities alongside dyslexia. The study found that dyslexia rarely appeared in isolation within the sample (see Table 3). A significant proportion of the sample reported being autistic (53.0%, *n* = 88), and/or ADHD (45.2%, *n* = 75). Dyspraxia (33.7%) and Irlen Syndrome (27.1%) were also relatively common. Smaller proportions reported dyscalculia (13.3%), pathological demand avoidance (7.2%), dysgraphia (6.0%) or developmental language conditions such as stuttering (3.6%).^1^ Very small numbers reported being non‐speaking or multimodal communicators, having Tourette's, an intellectual disability or global developmental delay. These data highlight that preferences around dyslexia labels are situated within broader neurodivergent identities for many participants, rather than being linked solely to dyslexia.

**TABLE 3:** Dyslexia and intersections of neurodiversity.

Additional neurodivergent identities		Percent	*n*
Autism		100%	166
	No	47.0	78
	Yes	53.0	88
ADHD		100%	166
	No	54.8	91
	Yes	45.2	75
Dyspraxia		100%	166
	No	66.3	110
	Yes	33.7	56
Irlen syndrome		100%	166
	No	72.9	121
	Yes	27.1	45
Dyscalculia		100%	166
	No	86.7	144
	Yes	13.3	22
Pathological demand avoidance (PDA)		100%	166
	No	92.8	154
	Yes	7.2	12
Dysgraphia		100%	166
	No	94.0	156
	Yes	6.0	10
Developmental language conditions, that is, stuttering		100%	166
	No	96.4	160
	Yes	3.6	6
Non‐speaking, semi‐speaking, multi‐modal communicator		100%	166
	No	97.6	162
	Yes	2.4	4
Tourette's		100%	166
	No	98.2	163
	Yes	1.8	3
Intellectual disability		100%	166
	No	98.8	164
	Yes	1.2	2
Global developmental delay		100%	166
	No	99.4	165
	Yes	0.6	1

*Note:* *n*, participant numbers.

## 7 Dyslexia, Identity and Quantitative Analysis

The data analysis in Table 4 demonstrates a clear hierarchy in participants' preferences for dyslexia‐related terminology. The term ‘dyslexic’ was the most positively received label. A total of 79.9% of participants reported that they preferred the term, compared with 14.0% who were neutral and 6.1% who rejected it. This distribution indicates a strong and widely shared preference for this identity‐first term across the sample. A similar but less pronounced pattern was evident for ‘dyslexic person’. While a majority still expressed positive views, the proportion who liked this term fell to 56.7%, with 27.4% reporting neutrality and 15.9% rejecting the term. This shift suggests greater ambivalence towards the term, despite its continued grounding in dyslexia‐specific identity. The increase in neutral and negative responses indicates that small linguistic differences within identity‐first language can meaningfully shape how labels are perceived. Responses to ‘person with dyslexia’ were more evenly distributed, reflecting that most participants were mixed in their views about adopting the term. Just over a third of participants reported liking this term (36.4%), while a comparable proportion were neutral (35.8%) and just under a third reported disliking it (27.9%). This suggests that person‐first language does not command strong endorsement within the sample and is experienced as acceptable by some participants but is undesirable by others.

**TABLE 4:** Preferred labels.

Terminology	Percent	*n*	Rank
Dyslexic	100%	164	M = 1.57
Dislike	6.1	10	SD = 1.1
Neutral	14.0	23	
Like	79.9	131	
Dyslexic person	100%	164	M = 2.64
Dislike	15.9	26	SD = 1.2
Neutral	27.4	45	
Like	56.7	93	
Person with dyslexia	100%	165	M = 2.76
Dislike	27.9	46	SD = 1.4
Neutral	35.8	59	
Like	36.4	60	
A person with a specific learning difficulty	100%	164	M = 4.33
Dislike	54.3	89	SD = 0.99
Neutral	25.6	42	
Like	20.1	33	
A person with a learning difficulty	100%	165	M = 5.40
Dislike	64.8	107	SD = 1.2
Neutral	18.2	30	
Like	17.0	28	
A person with a specific learning disability	100%	165	M = 5.69
Dislike	64.2	106	SD = 1.3
Neutral	20.6	34	
Like	15.2	25	
A person with a specific learning disorder	100%	166	M = 6.06
Dislike	70.1	115	SD = 1.2
Neutral	17.7	29	
Like	12.2	20	

*Note:* *n*, participant numbers.

Abbreviations: M, mean; SD, standard deviation.

In contrast, labels that framed dyslexia within broader categories of learning difficulty, disability or disorder were consistently rejected despite it being framed this way in educational practice. Over half of participants disliked a ‘person with a specific learning difficulty’ (54.3%), rising to 64.8% for a ‘person with a learning difficulty’. Similar levels of rejection were observed for a ‘person with a specific learning disability’ (64.2%) and ‘a person with a specific learning disorder’, which attracted the highest level of dislike overall (70.1%). Across these labels, fewer than one in five participants reported liking the term, indicating a clear distancing from language that generalises dyslexia into wider, deficit classifications. These findings demonstrate that preferences are not weakly held or randomly distributed. Instead, they reveal a clear gradient, with dyslexia‐specific identity‐based terms attracting the strongest support, person‐first language producing mixed responses and broader medicalised or deficit‐framed labels being widely disliked across the sample.

## 8 Dyslexia, Identity and Narrative Accounts

When exploring preferences concerning labelling in the qualitative data, similar themes were developed concerning positive constructions of personal identity. Across the dataset, participants consistently showed a preference for specific and non‐deficit‐based terminology, with dyslexia or dyslexic widely regarded as clearer and more acceptable than broader medicalised labels. Many participants emphasised that dyslexia is best understood as a difference rather than a deficit, and strongly rejected language that implied reduced intelligence, disorder or general learning incapacity. However, within the study, the term disability was often interpreted as deficit language, which is in direct conflict with the conceptualisation of disability that has emerged from the disability movement. However, this framing was not universal, and for some, dyslexia involved both strengths and disabling barriers, shaped by the social and institutional contexts in which it was experienced. As one participant stated, ‘not sure I see dyslexia as a disability’. Like disability, terms such as learning difficulty, learning disability and especially disorder were repeatedly described as misleading, stigmatising or inaccurate. Several participants explicitly rejected deficit framings on the grounds that they misrepresent both their abilities and their lived experience. One participant stated that:

Anything that says learning difficulty, disorder … is offensive as it gives the impression that you can't learn,

Another participant argued that such terms reflect a narrow interpretation of what it means to be dyslexic, stating, ‘I don't think of dyslexia as a disability or disorder.’ What is interesting about this quote is the association it makes between disorder and disability, where, from the participant's perspective, both are constructed as negative and pathologising forms of language.

The association between dyslexia and intelligence was a recurring concern, with participants noting that public misunderstanding persists. As one participant observed:

I was judged by an academic tutor as lazy because I have dyslexia. This did upset me …, as I always felt I was too “thick” to be studying at a degree level.

Therefore, because of existing stereotypes linking dyslexia to a perceived lack of intelligence, there was resistance to the deficit construction of dyslexia. In contrast, the use of dyslexia and dyslexic were frequently described as precise, factual and socially acceptable. Participants valued the specificity of these terms and their ability to communicate needs without over‐generalisation. One participant explained that:

I'm not a fan of the word disorder, sounds like I'm all over the place. And Dyslexic I think best describes what I have and is specific, the others seem too general to me,

Another commented that educational alternatives were necessary. This emphasis on directness reflects a broader preference for terminology that does not obscure or dilute meaning. Views on identity‐first versus person‐first language were more mixed, but notably pragmatic rather than ideological. Some participants strongly endorsed identity‐first language, arguing that dyslexia cannot be separated from the self, while others used each term interchangeably. One participant explained this, ‘To me “dyslexic person” suggests that dyslexia is a key part of who I am’, concluding that more general phrases like learning difficulties were unhelpful. Importantly, many participants stressed that context matters, with acceptability shaped by who is speaking, why the term is being used and whether reasonable adjustments are being discussed.

I feel the terminology use can be dependent on the setting, educational settings focus on the learning aspect, so the acknowledgment from staff of a learning disability, might prompt conscious teaching to assist more. But in the workplace, I want to focus on my strengths and not use such language.

A dominant theme across responses was rejection of the term ‘disorder’, which was described in explicitly emotional terms. Participants repeatedly framed disorder as pathologising, or dehumanising. For example, one participant stated bluntly:

I don't like disability and really hate disorder, condition is all right but I prefer specifying that I'm dyslexic.

Even among those who acknowledged that dyslexia is legally classified as a disability, there was resistance to applying disorder and disability to personal identity. For these participants, this led to separating dyslexia not only from the medical concept of disability but also from the disability movement more broadly. As one participant put it:

I don't want the ‘difficulty’ or disorder to be linked to me as a person. Yes, there are difficulties I experience but there are equally strengths.

Participants also expressed concern that broader educational terms, such as learning disability, are sometimes used in ways that blur distinctions with intellectual disability, which many felt was inaccurate and potentially harmful. This was articulated particularly strongly by these who worried that imprecise language could undermine credibility or misrepresent support needs. One participant explained that:

Learning disabilities … I think it is inaccurate to describe someone who only has one area of learning strongly impacted with such a broad term. It also implies to the general public Intellectual disability so if I rock up with a degree saying I'm learning disabled it's confusing and possibly quite insulting to those who are more globally learning disabled.

This concern contributed to a strong preference for dyslexia‐specific terminology when describing individual experiences. Finally, while some participants expressed comfort with umbrella concepts such as ‘neurodiverse’ or neurodivergent, this acceptance was generally conditional and secondary to dyslexia‐specific identification. Neurodiversity language was often valued for its non‐deficit framing, for example, because it ‘just says my brain works in a different way,’ but many participants viewed it as too broad or insufficiently descriptive for everyday use. Dyslexia remained the preferred label for clarity, self‐understanding and communication with others, even among those who accepted neurodiversity as a broader conceptual framework. The qualitative data show that participants overwhelmingly preferred dyslexia‐specific terminology over medicalised, deficit‐based or overly broad labels. Neurodiversity‐related language was generally acceptable but secondary, valued mainly for its positive framing rather than as a primary identity label.

## 9 Neurodivergence, Identity and Quantitative Analysis

Within the literature, there is very little data illustrating whether the dyslexic community prefer or use the terminology of neurodiversity to represent their identity. However, dyslexic people often have co‐occurring neurodivergences such as ADHD or autism (Brimo et al. 2021). The study therefore examined whether having a single diagnosis of dyslexia, or dyslexia that co‐occurred with other neurodivergences, influenced the use of terminology such as neurodiversity or neurodivergent. Within the data analysis, having a single diagnosis of dyslexia, alongside other co‐occurring neurodivergences such as dyspraxia, Irlen syndrome or dyscalculia, showed no significant difference in preferences towards neurodiversity. However, there was a significant difference when comparing preferences among dyslexic participants alongside ADHD and/or autism (*p* = 0.001; *p* = 0.002). In general, 66.9% of dyslexic participants accepted and used neurodivergent terminology (see Table 5). While awareness of the term neurodiversity was almost universal across the sample (96.4%; see Table 6), its adoption as a self‐identity varied markedly depending on whether dyslexia was experienced alone or alongside ADHD or autism.

**TABLE 5:** Dyslexic participants' use of the term ‘neurodiversity’.

Neurodiversity	Percent	*n*
Do you sometimes use the term ‘neurodiversity’	100%	166
Yes	66.9	111
No	30.7	51
Not sure	2.4	4

*Note:* *n*, participant numbers.

**TABLE 6:** Dyslexic participants' awareness of the term ‘neurodiversity’.

Neurodiversity	Percent	*n*
Heard of the term ‘neurodiversity’	100%	166
Yes	96.4	159
No	1.8	3
Not sure	1.8	3

*Note:* *n*, participant numbers.

Among participants with a dyslexia‐dominant identity, just over half described themselves as neurodivergent (57%), while a substantial minority did not (43%; see Tables 7 and 8). This indicates that, even within a highly informed sample, dyslexia alone does not lead to a uniform uptake of neurodivergent identity. Instead, identification as neurodivergent appears to remain contested or situational for a significant proportion of dyslexic adults. This group shows the greatest difference towards the neurodivergent label, despite near‐universal familiarity with the concept. In contrast, dyslexic participants and autism were significantly more likely to describe themselves as neurodivergent (Table 7). In this group, 79% identified as neurodivergent, compared with only 21% who did not (*p* = 0.002). This represents a substantial shift away from the analysis observed in the dyslexia dominant identity group. This difference suggests that the presence of autism alongside dyslexia is associated with a much stronger alignment with neurodivergent identity, rather than mere increased awareness of the term. A similar finding was evident among participants in Table 8 with dyslexia and ADHD. In this group, 82% of dyslexic participants and ADHD described themselves as neurodivergent, compared with just 18% who did not (*p* = 0.001). The consistency of this analysis across both dyslexia–autism and dyslexia–ADHD participants strengthens the claim that the terminology of neurodiversity is preferred by those with autism and ADHD rather than by those with a dyslexia dominant identity.

**TABLE 7:** Co‐occurring identities (autism).

		Dyslexia	Dyslexia and autism	Sig
Describe yourself as neurodivergent	Yes	*n* = 43	68	*p* = 0.002
		57.0%	79%	
	No	*n* = 33	18	
		43%	21%	

*Note:* *n*, participant numbers.

**TABLE 8:** Co‐occurring identities (ADHD).

		Dyslexia	Dyslexia and ADHD	Sig
Describe yourself as neurodivergent	Yes	*n* = 51	60	*p* = 0.001
		57%	82%	
	No	*n* = 38	13	
		43%	18%	

*Note:* *n*, participant numbers.

These findings indicate a mixed relationship between diagnostic differences and neurodivergent self‐identification. Participants with a dyslexia‐dominant identity were the least likely to adopt the neurodivergent label, while those with dyslexia plus autism or ADHD were substantially more likely to do so. Importantly, this finding cannot be explained by lack of exposure to neurodiversity terminology, given the uniformly high levels of awareness across the sample. Instead, the data suggest that a neurodivergent identity is more readily adopted when dyslexia is experienced alongside co‐occurring neurodivergent identities such as ADHD or autism. The presence of autism or ADHD alongside dyslexia may make neurodivergence more relevant across multiple domains of life, increasing the relevance and perceived accuracy of the neurodivergent label. Therefore, they are adopted differently depending on how individuals locate their differences in relation to social norms, preferences and lived experiences.

## 10 Neurodivergence, Identity and Narrative Accounts

Similar arguments were developed from the qualitative data, suggesting that participants are more likely to adopt a neurodiversity label when dyslexia co‐occurs with other neurodivergences. These findings reveal how neurodiversity‐related terminology is understood, accepted and used. While both groups strongly rejected deficit‐based language, their positioning towards neurodiversity and neurodivergent terminology differed significantly. Participants with a dominant identity of dyslexia overwhelmingly favoured dyslexia‐specific terminology and were cautious about adopting neurodiversity language as a primary identity. Across the dyslexia–dominant group, dyslexia and dyslexic were valued for their clarity, particularly in educational and professional contexts. These terms were seen as sufficiently precise in explaining their identities, rather than drawing on less specific neurodivergent terminology.

The meaning and who falls under neurodivergent is sometimes unclear. As someone with dyslexia I would not class myself as neurodivergent. I also often hear some people wrongly using neurodivergent to solely to mean autistic people.

Neurodiversity‐related terms were treated more ambivalently. While some participants acknowledged that neurodiverse or neurodivergent could feel far less deficit‐focused than disorder or learning disability, many described these terms as too broad and unclear. Several participants explicitly stated that they did not feel entitled to use neurodivergent language in the absence of additional diagnoses.

I don't feel ‘neurodivergent’ enough to use that word, I only have dyslexia and not multiple other learning/social difficulties.

This distinction suggests that participants are engaging in a careful negotiation of language rather than rejecting neurodiversity outright. Accounts indicated that dyslexia was understood as a specific lived experience that did not automatically translate into a broader neurodivergent identity, reflecting a preference for diagnostic specificity, particularly in relation to identity. Notably, some participants expressed uncertainty about whether they were entitled to use the term ‘neurodivergent’, suggesting that the label is perceived as bounded and unevenly available. This points to underlying power dynamics within neurodiversity discourse, where forms of epistemic authority shape who is recognised as legitimately belonging within the category. At the same time, the discourse does not dismiss neurodiversity language entirely. Instead, it is repositioned as functionally useful in collective or policy contexts rather than as a primary marker of selfhood, as one participant states:

As an accessibility professional, I often discuss people's needs and requirements with managers, medical professionals and families. What I tend to find is that when someone who is perceived to be in authority used language such as learning disability, people judge this to mean a person is incapable of learning in any form. However, when you say someone is neurodiverse, or thinks differently, then people are more likely to be open to approaching learning in a different way that suits the individual.

Overall, dyslexia‐dominant participants positioned neurodiversity language as optional, secondary to dyslexia‐specific terminology and sometimes as misaligned with their lived experience. In contrast, dyslexic participants alongside other neurodivergent identities were far more likely to embrace neurodiversity‐related terminology as meaningful, affirming and practically useful. Within this group, neurodivergent and neurodiversity were frequently described as capturing the complexity of multiple diagnoses, reducing the need to repeatedly list co‐occurring neurodivergences and resisting medicalised deficit framings.

I don't like most language, I prefer just the acknowledgement of natural differences, even neurodiversity, surely diverse means a range of being, rather than some people being typical and some people being diverse.

For participants, neurodiversity was framed as an identity through which they reclaimed ownership over their own experiences. Rather than accepting deficit or clinical descriptions, the term is used to construct a more holistic narrative of self that illustrates difference rather than pathology. In this sense, neurodiversity becomes both an identity and a political stance, challenging the dominance of professional discourse. The emphasis on diagnosis, power and labelling suggests that language is understood not as neutral, but as constitutive of social hierarchy and control.

neurodiverse is a holistic term that simply says this person thinks/or acts differently to what “most” people will do. This means that people are not prejudged and can explain their difference in a way that is suitable to them, if necessary.

Participants with multiple neurodivergent identities were less likely to question whether they were ‘entitled’ to a neurodivergent identity. Instead, they focused on how such language could be misused or imposed by professionals without listening to the neurodivergent community. Neurodiversity was treated as a collective identity, but one that required respect for internal diversity and self‐identification. While neurodiversity is embraced as a shared political and social identity and movement, participants remain alert to the ways in which practitioners may appropriate or regulate the terminology in ways that dilute its political intent. The concern is not with the label itself, but with who has the authority to define, operationalise and apply it within an institutional setting. From a participant perspective, neurodiversity becomes a site of self‐identity, where legitimacy is grounded in lived experience rather than professional expertise. As one participant suggests:

I use neurodiverse in reference to myself when I am talking about some of the issues that cut across the different conditions, such as executive function, literalism, memory problems.

These responses suggest that participants with more than one neurodivergence engaged with neurodivergent terminology in a more pragmatic manner than the dyslexia‐dominant group. Rather than questioning the legitimacy of the label itself, they describe using neurodiversity language to refer to cognitive characteristics that cut across different co‐occurring neurodivergences. In this sense, the term functions as a practical linguistic shorthand that allows individuals to communicate shared experiences across diagnostic categories. At the same time, participants also demonstrate an awareness of the social and political dynamics surrounding the terminology. While some dyslexia‐dominant participants recognise themselves as neurodivergent, they remain cautious about adopting the label fully due to its perceived association with particular communities. In some cases, this caution appeared to involve forms of boundary‐making, where participants distanced themselves from groups more commonly associated with the term. This may reflect underlying hierarchies and stigma within neurodiversity discourse, shaping how individuals position themselves in relation to broader neurodivergent identities.

I realise I am neurodivergent but don't use the term as it has largely been adopted by the ‘autistic community’.

As one participant explained, although they understand themselves as neurodivergent, they avoid using the term due to social stereotypes which they feel dissociate the label from the dyslexic community. This highlights how the adoption of neurodiversity language is shaped not only by personal identification but also by perceptions of community ownership and the boundaries of collective identity. For participants with multiple neuro‐identities, neurodiversity operates both as a useful umbrella concept and a contested identity category. While the terminology enables individuals to articulate shared experiences across neurodivergent identities, its use remains negotiated through ongoing debates about representation, legitimacy and the right to self‐identify with a label.

## 11 Discussion

The findings demonstrate a clear preference for identity‐first, dyslexia‐specific language alongside a rejection of broader deficit‐based or medicalised labels. Similar findings were reported in an Australian study by Anthony et al. (2025), in which dyslexic adults, parents and professionals favoured specific terms such as dyslexia over broader labels such as learning disability and deficit‐based terminology. This convergence suggests that preferences for dyslexia‐specific language and resistance to deficit labelling extend across national contexts. Situated within the history of the disability movement, these findings are not merely about linguistic preference but about identity politics and control over meaning (Oliver 2009; Barnes 2019). Disability studies has long shown that labels are embedded within institutional practices that shape recognition, access to resources and the construction of identities (Barnes 2019). The preference of dyslexia as a label suggests that participants do not experience this term as pathologising. Instead, it functions as a community‐accepted label that affirms dyslexia as a reclaimed, either neutral or positive, identity. This preference does not reflect an uncritical embrace of diagnosis. Rather, it aligns with the suggestion that impairment‐specific labels can operate as socially mediated resources within a disabling society (Shakespeare and Watson 2023). For many participants, dyslexia appears to provide recognition and legitimacy without collapsing the label into a solely deficit‐based or overly broad description. Diagnosis is not uniformly experienced as stigmatising (Macdonald 2010, 2013). This is because stigma may precede and operate independently of the label itself. Many dyslexic people report being stigmatised because of their literacy abilities, which predated diagnosis (Macdonald 2010, 2013; Odegard and Dye 2024). Thus, the reclaiming of dyslexia reflects a re‐conceptualisation of impairment, shifting from the notion of ‘incapacity’ towards the concept of difference or diversity, echoing broader political constructions of neurodivergent identities (Odegard and Dye 2024).

In contrast, the rejection of terms such as learning difficulty, specific learning difficulty and specific learning disorder reflects resistance to homogenising classifications that collapse dyslexia into categories associated with broad learning deficits or dysfunctions (Campbell 2013). Therefore, these terms attracted the highest levels of negativity, suggesting experiences of misrecognition and, in some cases, reinforcing stigmatising notions of dyslexia (Riddick 2000; Macdonald 2010). What was surprising in the findings was that a significant proportion of dyslexic participants constructed the notion of disability as a deficit term, similar to difficulty or disorder. This reflects a tension between disability studies theory, public and professional discourse and lived experience (Oliver 2009; Barnes 2019). While participants rejected pathologisation in relation to dyslexia, some appeared to accept more individualised understandings when engaging with the label of disability. This ambivalence points to the influence of dominant narratives and power relations in shaping how disability is understood and negotiated. Consequently, the interpretation of dyslexia as a difference or variation was more closely aligned with the interpretation presented by the neurodiversity movement (Macdonald 2019; Chapman and Carel 2022). This supports the argument that resistance to deficit labelling is shaped by resistance to medicalisation, which for many participants does not reflect their lived experience (Shakespeare 2013). In this respect, the findings support neurodiversity politics, which challenge professional dominance and medicalisation that, from the participants' perspective, do not reflect the reality of dyslexia (Ne'eman and Pellicano 2022; Odegard and Dye 2024).

Yet the adoption of a neurodivergent identity was somewhat mixed in the study (Runswick‐Cole 2014; Jones and Orchard 2024). Participants reported widespread familiarity with neurodiversity, but unequal adoption of the concept as an identity indicated a selective engagement with neurodiversity language. Furthermore, although pathologisation was rejected, dyslexia remained the preferred term across groups. The adoption of neurodiversity as an identity was somewhat mixed, depending on whether participants primarily identified as dyslexic or whether dyslexia formed part of an identity alongside ADHD, autism or both. This selective acceptance reflects a complex political position, participants embrace neurodiversity as a label of solidarity and sometimes as a discourse of empowerment, while maintaining diagnostic specificity to secure recognition and material support (Riddick 2000; Macdonald 2010, 2013; Odegard and Dye 2024). However, this depended on how dyslexia intersected with other neurodivergent identities. These findings therefore illustrate the political complexity of labelling within the dyslexic community (Macdonald 2013, 2019). Participants' preferences do not signal a simple shift from medical to social models, nor a straightforward rejection of impairment labels in favour of neurodiversity terminology (Macdonald 2019; Odegard and Dye 2024). Instead, they reveal an ongoing negotiation between identity, impairment and institutional power. Impairment‐specific labels, that is, dyslexia, remain politically and materially significant. Ultimately, terminology is not a neutral technical matter but a political act (Campbell 2013; Kirby 2019). It shapes recognition, access to support, and the boundaries of collective identity. For participants in this study, dyslexic represents a claim to self‐identity grounded in lived experience and diagnostic specificity, while broader deficit labels are rejected as misunderstanding of neurodivergence.

## 12 Conclusion

This article examined how dyslexic adults understand and negotiate the language used to describe dyslexia and neurodiversity. The findings demonstrate that terminology is neither neutral nor interchangeable, but socially and politically important. Participants showed a strong preference for dyslexia‐specific, identity‐first language and consistently rejected broader deficit terminology. These preferences reflect concerns about accuracy, stigma and misrecognition, as well as acknowledging the broader impacts of discrimination and exclusion. For many, identity‐first language was experienced as affirming and precise, while person‐first and medicalised labels were viewed as discriminatory. While awareness of neurodiversity discourse was widespread, identification with neurodivergent language varied according to diagnostic complexity. Dyslexic participants were more ambivalent than those with co‐occurring autism or ADHD, suggesting that neurodivergent identity is shaped by intersectional factors concerning other neurodivergent identities rather than by ideological commitment alone. Neurodiversity language was valued for its non‐deficit framing, yet it was often regarded as too broad where dyslexia was experienced as a discrete form of being. The study illustrates the importance of centring dyslexic voices in debates about terminology. Language choices made by institutions carry material consequences for recognition, legitimacy and access to support. The rejection of deficit labelling reflects resistance to misrecognition rather than rejection of diagnosis. Dyslexic adults are not opposed to labelling; they are selective about labels that accurately and meaningfully represent their lives. The article therefore calls for a reflexive and contextual approach to terminology. Labels should be understood as situated social tools, shaped by power, policy and lived experience. Attending to how dyslexic people negotiate language in practice offers a more grounded basis for research, policy and inclusive practice. This advances debates within dyslexia scholarship beyond professional definitions produced in laboratories or classrooms to the material and political impact that labels have on the dyslexic community.

## 13 Strengths and Limitations

This study combines quantitative and qualitative data to provide detailed insight into dyslexic adults' perspectives on terminology, centring participant voices. However, convenience and snowball sampling produced a predominantly White, female, highly educated and largely England‐based sample. Nearly three‐quarters held an undergraduate degree or higher qualification. Experience of higher education may increase familiarity with diagnostic terminology and the use of dyslexia‐specific labels to secure recognition and reasonable adjustments. This may help explain the value attached to diagnostic specificity within the sample. Adults with fewer qualifications, different educational experiences or less access to assessment and institutional support may have different relationships with these labels. The limited ethnic diversity restricts consideration of how cultural understandings of disability, racialised experiences and inequalities in access to diagnosis may shape language preferences. The predominance of female participants and those living in England also limits the extent to which the findings capture experiences across genders and different national education and support systems. Recruitment through neurodivergence‐related organisations, researchers' networks and social media may have disproportionately reached people already engaged with neurodivergent communities. The high awareness of neurodiversity terminology should therefore not be assumed to reflect awareness among dyslexic adults more broadly. These findings represent situated preferences rather than a consensus across the dyslexic community. Online participation may have presented barriers for some people with literacy or digital access difficulties, while qualitative responses varied in depth and could not be explored through follow‐up questioning. Co‐occurring neurodivergences complicate the attribution of preferences specifically to dyslexia. Finally, the cross‐sectional design cannot establish how preferences change through experiences of diagnosis, support or community engagement. Further research with more diverse samples would help assess the extent to which these patterns are shared.

## Conflicts of Interest

The authors declare no conflicts of interest.

## Data Availability

Data sharing not applicable to this article as no datasets were generated or analysed during the current study.

## Endnotes

^1^
Participants could select all neurodivergent identities that applied.
